# Transmission of Schmallenberg Virus during Winter, Germany

**DOI:** 10.3201/eid1910.130622

**Published:** 2013-10

**Authors:** Kerstin Wernike, Mareen Kohn, Franz J. Conraths, Doreen Werner, Daniela Kameke, Silke Hechinger, Helge Kampen, Martin Beer

**Affiliations:** Friedrich-Loeffler-Institut, Insel Riems, Germany (K. Wernike, M. Kohn, D. Kameke, S. Hechinger, H. Kampen, M. Beer);; Friedrich-Loeffler-Institut, Wusterhausen, Germany (F.J. Conraths);; Leibniz Centre for Agricultural Landscape Research, Müncheberg, Germany (D. Werner)

**Keywords:** Schmallenberg virus, sheep, winter, transmission, *Culicoides* biting midges, viruses, Germany

**To the Editor:** Schmallenberg virus (SBV), an orthobunyavirus, emerged in northern Europe in 2011 (*1*). SBV infection causes transient fever, diarrhea, and a reduced milk yield in adult ruminants but, most notably, stillbirths and severe malformations in lambs and calves (*2*). Insect vectors play an essential role in transmission; the viral genome has been detected in various field-collected biting midges (*Culicoides* spp.) (*3*,*4*).

During autumn 2012 and winter 2012–2013, blood samples were taken at several times from individual sheep on a farm located in the German federal state of Mecklenburg–Western Pomerania. The farm is surrounded by agricultural fields and meadows. Approximately 1,000 ewes and their lambs, a dog, and some cats were kept on the farm; most of the animals are outdoors year-round. Only dams with >2 lambs are housed in open stabling in December and January. The dung is regularly cleared away and stored ≈10 m from 1 of the stable entrances. Repellents or insecticides were not applied in the monitored period. Blood samples were taken in September 2012 and in January and February 2013 and analyzed by an SBV-specific real-time quantitative reverse transcription PCR (RT-qPCR) (*5*) and by an SBV antibody ELISA (ID Screen Schmallenberg virus Indirect; IDvet; Montpellier, France) by using the recommended cutoff of 50% relative optical density as compared with the positive control (sample-to-positive ratio [S/P]).

In September 2012, blood samples from 60 sheep tested negative by the SBV antibody ELISA. Moreover, fetal malformations of the brain, spinal cord, or skeletal muscle, which might have suggested a previous SBV-infection of the dam, were not observed during the lambing season in December 2012.

On January 10, 2013, blood samples were taken from 15 sheep that had not previously been tested; samples from all animals tested negative by ELISA. However, 4 sheep (S01–S04) tested positive by RT-qPCR (quantification cycle values: S01: 31.6, S02: 39.9, S03: 37.6, and S04: 34.9). Four weeks later, antibodies against SBV could be detected. Each of the PCR-positive blood samples was injected into 2 adult type I interferon receptor-knockout mice on a C57BL/6 genetic background. Both mice that had received blood samples of sheep S01 were seropositive after 3 weeks (S/P: 207.0 and 207.2), which demonstrates the presence of infectious virus in the inoculated blood. Assuming that viral RNA remains in the blood for just a few days, as reported after experimental infection with SBV (*1*,*6*), the sheep tested in this study had most likely been infected in early 2013. During this period, the lowest temperatures rose above 5°C for several consecutive days, with a maximum of ≈9°C (Figure, panel A). Within this brief interval, when the temperature was higher, some biting midges (*Culicoides* spp.) become active (*7*). Indeed, at the end of January, a single female biting midge (Obsoletus complex) was caught in a trap equipped with ultraviolet light; the midge tested negative by the SBV-specific RT-qPCR.

**Figure:**
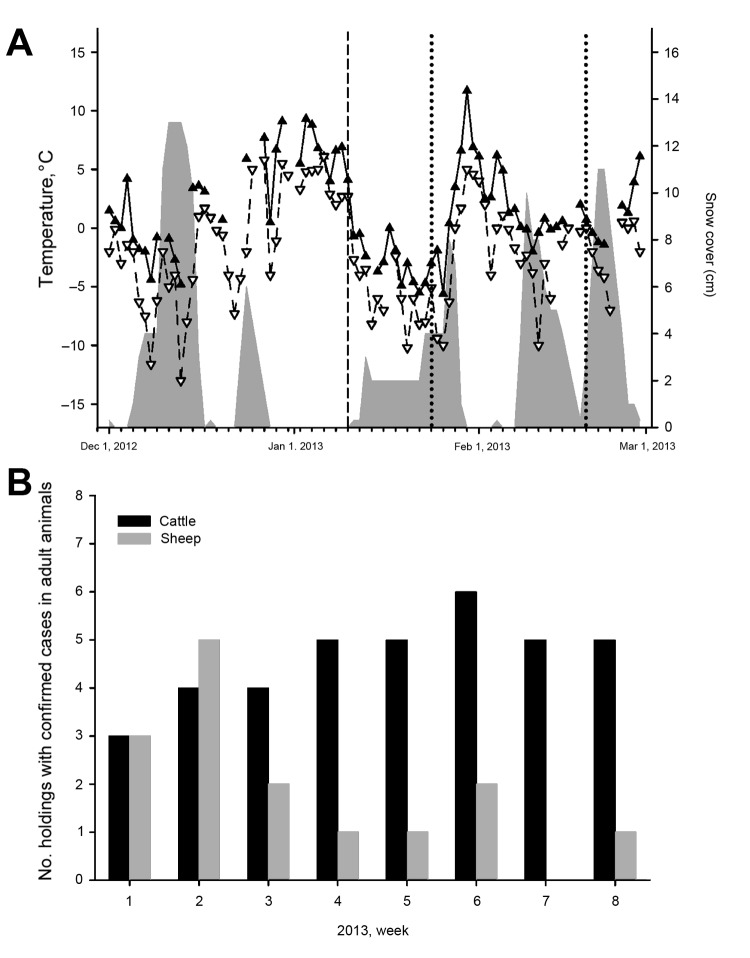
Results of analysis of samples from sheep and cattle for Schmallenberg virus (SBV), Germany, 2012–2013. A) Climate data and sampling. The maximum temperatures are shown with filled triangles and a solid line and the minimum temperatures with unfilled triangles and a broken line. Snow cover is symbolized by a gray area. The dashed line represents the day of the detection of SBV genome in 4 sheep. Further sampling days are marked by dotted lines. B) PCR-confirmed Schmallenberg virus infections in adult cattle (black bars) or sheep (gray bars) in Germany during January 1–February 20, 2013.

On January 23 and February 20, 2013, blood samples were taken from 90 sheep that had not previously been tested (Figure, panel A). A viral genome was not detected in any animal at any time. However, antibodies were detectable in 9 animals on the first sampling day. In 2 additional sheep, the S/P was in the inconclusive range; 1 of the animals tested positive after 4 weeks. In the remaining 79 sheep, no SBV antibodies could be detected; after 4 weeks, 76 sheep still tested negative by ELISA. However, the S/P of 1 sheep had increased to the inconclusive range, and 2 sheep were seropositive. Because antibodies may be detectable 10 days–3 weeks after experimental infection for the first time (*8*), the presumed period of infection was between mid-January and mid-February. At this time, the highest temperatures again rose above 6°C for a few days (Figure, panel A).

Although the within-herd seroprevalence was >90% in ewes after confirmed or suspected SBV infection in 2011 (*9*), in this study, conducted during the cold season, only 12 (13%) of 90 tested sheep were positive by ELISA. Three animals seroconverted between mid-January and mid-February. Thus, SBV transmission appears to be possible at a low level, most likely because of the low activity of the involved insect vectors.

In addition to the SBV cases found on the sheep holding in Mecklenburg–Western Pomerania, an additional 52 confirmed SBV cases (defined as virus detection by qRT-PCR or isolation in cell culture) in adult ruminants were reported to the German Animal Disease Reporting System from January 1 through February 20, 2013 (Figure, panel B). Most affected animal holdings were located in Bavaria, but cases were also reported from Thuringia, Saxony, Brandenburg, Mecklenburg–Western Pomerania, Hesse, and Lower Saxony. In conclusion, transmission of SBV by hematophagous insects seems possible, even during the winter in central Europe, if minimum temperatures rise above a certain threshold for several consecutive days.
